# A Mobility Model for a 3D Non-Stationary Geometry Cluster-Based Channel Model for High Speed Trains in MIMO Wireless Channels

**DOI:** 10.3390/s222410019

**Published:** 2022-12-19

**Authors:** Eva Assiimwe, Yihenew Wondie Marye

**Affiliations:** African Railway Center of Excellence, Addis Ababa Institute of Technology, Addis Ababa University, King George VI St., Addis Ababa 1000, Ethiopia

**Keywords:** 3D GBSM, Non-WSS channel, dynamic speed, stationary interval, cluster model, enhanced Gauss–Markov, HST

## Abstract

During channel modeling for high-mobility channels, such as high-speed train (HST) channels, the velocity of the mobile radio station is assumed to be constant. However, this might not be realistic due to the dynamic movement of the train along the track. Therefore, in this paper, an enhanced Gauss–Markov mobility model with a 3D non-stationary geometry based stochastic model (GBSM) for HST in MIMO Wireless Channels is proposed. The non-isotropic scatterers within a cluster are assumed to be around the sphere in which the mobile relay station (MRS) is located. The multi-path components (MPCs) are modeled with varying velocities, whereas the mobility model is a function of time. The MPCs are represented in a death–birth cluster using the Markov process. Furthermore, the channel statistics, i.e., the space-time correlation function, the root-mean-square Doppler shift, and the quasi-stationary interval, are derived from the non-stationary model. The model shows how the quasi-stationary time increases from 0.21 to 0.451 s with a decreasing acceleration of 0.6 to 0.2 m/s${}^{2}$ of the HST. In addition, the impact of the distribution of the angles on the channel statistics is presented. Finally, the simulated results are compared with the measured results. Therefore, there is a close relationship between the proposed model and the measured results, and the model can be used to characterize the channel’s properties.

## 1. Introduction

The space–air–ground–sea integrated network (SAGSIN) will focus on more diversified and dynamic communication scenarios, including vehicle-to-vehicle (V2V), high-speed train (HST), unmanned aerial vehicle (UAV), satellite, and maritime communications [1]. This is the wireless communication network’s vision for the B5G/6G (Beyond 5G/6G) era. Therefore, accurate and user-friendly channel models that can accurately mimic the underlying characteristics of the B5G/6G channels are essential for the successful design of this communication system. Channel modeling in these dynamic channel scenarios is essential for the evaluation and performance of a wireless communication system before and during implementation. The channel model represents how MPCs in a non-stationary wireless channel propagate in actual scattering situations [2,3]. This is crucial for assessing the effectiveness of communication systems. Some 2D geometry-based stochastic models (GBSM) with non-stationary characteristics have been presented in [4,5,6,7]. However, in 5G and beyond, elevation angles need to be considered. In [8,9,10,11], a 3D channel model for a high-speed train was proposed, and its channel statistics were derived. However, in [8,11], some of these channel models were based on ray tracing, and they only considered distinct dimensions for particular HST environments. In [10], the MPC’s non-stationarity was based on tapped delay line, and yet a clustered channel model provides better insights about the channel characteristics. A cluster in this context is a collection of MPCs with identical delay, power, and angle characteristics. The ability to extract intra-cluster and inter-cluster statistics is typically provided by a cluster-based channel architecture, since statistical models with a few parameters, such as Laplacian or Gaussian distributions, can frequently capture the intra-cluster features. This provides more intuitive insights, and the model can further be compared with existing standard channel models.

In [12,13], the term "BS-Visibility Region (VR)" was used to describe the partially visible nature of clusters. The visibility nature of clusters has been mimicked using the birth–death cluster, which is also known as the Markov process. This has been documented in several studies, including [4,14]. In the B5G/6G channel model development, it is generally necessary to find a technique to effectively represent the exact channel characteristics. The non-stationary channel characteristics have to be captured in both domains of space and time [14]. Several channel features in 5G systems are intended to be represented by a GBSM, which is also termed a generic 5G channel model (MG5GCM) [15]. The model is capable of supporting numerous communication scenarios based on the general model structure, but the direction angles (azimuth and elevation), the travel times between the TX/RX, and the scattering components were determined separately. In [4], the model disregarded the non-stationary features in the frequency domain and could only change in the time and array axes. It is challenging for the model to attain spatial consistency because the angles and delays indirectly determine the positions of scatterers.

Some non-stationary GBSMs which have been proposed for HST channels and V2V channels have some characteristics in common, such as high Doppler shifts and temporal non-stationarity [16]. However, these cannot be generalized to all channels with the Doppler effect. For HST conditions where the train velocity could reach 350 km/h, widely used standard channel models are WINNER I [17], WINNER II [18], and IMT-Advanced channel models [19]. The models discussed above are only two-dimensional (2D) and can only be used in situations when the transceiver and scatterers are sufficiently apart. In addition, some of these models were generated using the temporal wide-sense stationary (WSS) assumption, and the cluster dynamics in the time domain were overlooked. By considering the time-varying angles and cluster dynamics, the HST channel model in [2] was proposed based on the IMT-Advanced channel model; however, this is a 2D model, yet elevation angles are important especially when there are ground reflection rays. Although the channel characteristics in B5G/6G systems are intended to be captured by the GBSM [3], the suggested models are generalized for all the dynamic communication scenarios. This can be seen in some of the recent models presented in [12,14,17,20,21].

The elliptical GBSM for the HST channel model which considered varying movement speed and direction was proposed in [4,22]. However, the model was two-dimensional (2D) and can only be used in situations when the transceiver and scatterers are significantly separate from each other. A non-stationary 3D deterministic model based on ray tracing for the HST channel model was proposed in [23]. However, the model could only be utilized for tunnel environments. A 3D GBSM based on a tapped delay line wireless channel model for several HST environments was proposed in [17]. The model’s validity depends on the parameters of the models developed from the viaduct and cutting environments acquired during ray tracing. However, the whole procedure results in a high level of computational complexity.

The HST undergoes different trajectories of acceleration and deceleration. This happens during the taking off, change in velocity as it goes through different environments, and stopping at the station. On the other hand, beam misalignment due to dynamic vehicle traffic tends to lower quality-of-service (QoS) performance [24]. Given this trajectory, the HST dynamic velocity and motion direction are usually ignored during channel modeling. For channel models situated in 5G and beyond, mobility modeling specifications in environments with the Doppler effect have to be considered. The d hoc network simulations frequently employ the Gauss–Markov mobility model [25]. This model has lately been used to describe the UAV channel due to its accessibility and efficiency. However, since accelerating or decelerating operations, such as the starting and stopping of HSTs, cannot be modeled using the Gauss–Markov mobility model, an enhanced Gauss–Markov mobility model from [26] was used in this research work to feature the acceleration and deceleration characteristics. The enhanced Gauss–Markov mobility model is simple and available. In this paper, a GBSM for a 5G HST dynamic communication system is proposed. The model was applied in the open-case scenario of the HST. The effect of scatters on the environment was also studied. The primary contributions of this research paper are:

1. A 3D, mobile and non-stationary cluster-based GBSM with scatterers located around the moving MRS is proposed.
2. The HST’s mobility is described by the enhanced Gauss–Makorv mobility model incorporating acceleration.
3. The death–birth Markov model is used to model the cluster MPCs.
4. The channel statistics, i.e., the local space-time correlation function (ST-CF), the root-mean-square Doppler shift spread, and the quasi-stationary intervals, are derived.
5. The simulated results of the proposed model are compared with the measured results.

## 2. A Mobility Model for a 3D, Non-Stationary Cluster and Geometry-Based Channel Model

**Figure 1:**
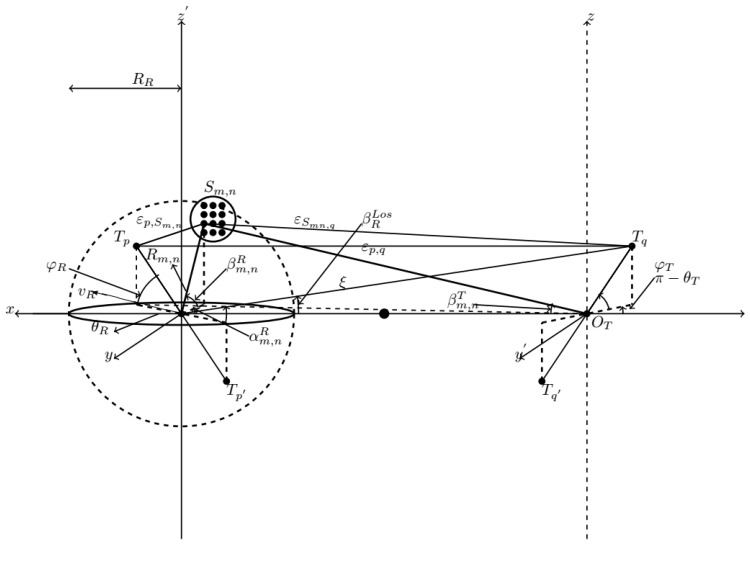
A 3D mobility non-stationary cluster GBSM for HST channels.

In this study, the channel impulse response (CIR) is derived from the proposed 3D non-stationary GBSM shown in Figure 1. The sum of sinusoid (SoS) simulation method in [27] corresponding to the model is derived. The MIMO channel with non-isotropic scatterers is considered. The CIR of the complex fading envelope of the MIMO channel has $\left[M_{T}\times M_{R}\right]$ matrix. The time-variant CIR ${\left[h_{pq}\left(t,\tau \right)\right]}_{M_{T}\times M_{R}}$ is a superposition of the sum of $h_{pq}^{Los}\left(t,\tau \right)$ and $h_{pq}^{NLos}\left(t,\tau \right)$, representing the LOS component and NLOS component, respectively. In this case, the BS antenna elements are P(p=1,2,…,P), and the antenna elements of the moving MRS are q(q=1,2,…,Q). There are $M_{T}$ omni-directional antennae on the BS and $M_{R}$ omni-directional antennae on the MRs. The MRs is located at the top roof of the train.

These parameters from Table 1 are considered for the transmission link at any time instant *t* from the transmitting antenna $T_{p}$ to $R_{q}$. By considering independent components of Los and NLoS, the following formula can be used to get the time-variant channel impulse response (CIR). In this work, the CIR of the $n^{th}$ cluster is considered to be a Gaussian process that follows the Lindeberg–Levy theorem. By considering this, the CIR equation is deduced to have a power variance of $P_{n}\left(t\right)/2$, with the expectation of 0. The condition is that the cluster number (N) tends to infinity. $P_{n}\left(t\right)$ denotes the normalized power which is received on the $n^{th}$ cluster, whereas the carrier wavelength is denoted by λ. The following equations are used to describe the two components ie.,

(1) $$h_{pq}\left(t,\tau \right)=h_{pq}^{Los}\left(t,\tau \right)+h_{pq}^{NLos}\left(t,\tau \right),$$

where

(2a) $$h_{pq}^{Los}\left(t,\tau \right)=\sqrt{\frac{K(t)}{K(t)+1}}e^{-j2\pi \varepsilon_{p,q}\left(t\right)/\lambda }e^{j2\pi f_{D}^{Los}\left(t\right)t}\times \delta \left(\tau -\tau_{Los}\left(t\right)\right)$$

(2b) $$h_{pq}^{NLos}\left(t\right)=\sqrt{\frac{1}{\left(K(t)+1\right)}}\sum_{n=1}^{N(t)}\lim_{M(t)\to \infty }\sqrt{\frac{P_{n}\left(t\right)}{M(t)}}\times \sum_{m=1}^{M(t)}e^{-j2\pi (\varepsilon_{p,S_{nm}}\left(t\right)+\varepsilon_{S_{nm},q})/\lambda }\times e^{j2\pi f_{nm,D(t)}t}\times e^{j\phi_{n,m}\left(t\right)}\times \delta \left(\tau -\tau_{n,m}\left(t\right)\right)$$

In this case, $\tau_{Los}\left(t\right)$ is the propagation delay of the LOS component, and $\tau_{n,m}\left(t\right)$ designates the propagation delay of the $m^{th}$ resolvable subpath in the $n^{th}$ cluster. The phase angle $\phi_{m,n}\left(t\right)$ is randomly distributed with a uniform distribution over [−π,π]. The direct link between $T_{x}$ and $R_{x}$ is $T_{p}\to R_{q}$ which is the wave traveling distance denoted by $\varepsilon_{p,q}\left(t\right)$ of LOS Component. The link through the $S_{n,m}\left(t\right)$ scatterer is $\varepsilon_{p,S_{n,m}}\left(t\right)+\varepsilon_{S_{nm,q}}\left(t\right)$, which represents the of the path distance from the $p^{th}$ antenna element to the $S_{n,m}$ scatterer and the path distance from the $S_{n,m}$ scatterer to the $q^{th}$ antenna element. The derivations of both travel paths are as follows.

The derivation of the travel-distance terms is written as

(3a) $$\varepsilon_{pq}\left(t\right)=\xi -\frac{\delta_{R}}{2\xi }\left[\frac{\delta_{T}}{2}\sin\varphi_{T}\sin\varphi_{R}-Q\cos\varphi_{T}\cos\varphi_{R}\right]$$

(3b) $$\varepsilon_{p,S_{n,m}}\left(t\right)=\xi_{S_{n,m}}\left(t\right)-\frac{\delta_{T}}{2\xi_{m_{1,1}}}\left[R_{n,m}\sin\beta_{n,m}^{R}\left(t\right)\sin\varphi_{T}+Q\cos\varphi_{T}\cos\left(\alpha_{n,m}^{R}-\theta_{T}\right)\right]$$

(3c) $$\varepsilon_{S_{nm,q}}\left(t\right)=R_{n,m}-\frac{\delta_{R}}{2}\left[\sin\beta_{n,m}^{R}\left(t\right)\sin\varphi_{R}+\cos\beta_{n,m}^{R}\left(t\right)\cos\varphi_{R}\cos\left(\theta_{R}-\alpha_{n,m}^{R}\right)\left(t\right)\right]$$

where $\xi \approx Q\approx D-\frac{\delta_{T}}{2}\cos\varphi_{T}\cos\theta_{T}$, $\xi_{n,m}\left(t\right)=\sqrt{Q_{n,m}{\left(t\right)}^{2}+R_{n,m}^{2}\sin^{2}\beta_{R}^{n,m}\left(t\right)}$, $Q_{n,m}\left(t\right)\approx D+R_{n,m}\cos\beta_{n,m}^{R}\left(t\right)\cos\alpha_{n,m}^{R}\left(t\right)$.

We can deduce the following relation seen between AoDs and AoAs of the SB rays as from the sphere model: $\alpha_{\left(n,m\right)}^{T}\left(t\right)\approx \frac{R_{n,m}}{D}\sin\alpha_{n,m}^{R}\left(t\right)$, $\beta_{n,m}^{T}\left(t\right)\approx \arccos\left(\frac{D+R_{n,m}\cos\beta_{n,m}^{R}\left(t\right)\cos\alpha_{n,m}^{R}\left(t\right)}{\xi_{(n,m}\left(t\right))}\right)$. The Doppler shifts of the $m^{th}$ LOS sub-path within the $n^{th}$ cluster are represented by $f_{D}^{LoS}\left(t\right)$ and $f_{nm,D}\left(t\right)$ in (2a) and (2b), respectively. The maximum Doppler shift resulting from the moving $R_{x}$ is $f_{R,max}=v_{R}/\lambda$.

(4a) $$f_{D}^{Los}=f_{max}t\cos\left(\alpha_{R}^{Los}-\gamma_{R}\right)\cos\beta_{R}^{Los}$$

(4b) $$f_{\left(nm\right)}^{D}=f_{max}t\cos\left(\alpha_{n,m}^{R}\left(t\right)-\gamma_{R}\right)\cos\beta_{n,m}^{R}\left(t\right)$$

The cluster sub-path number grows towards infinity. Thus, the latency difference between a cluster’s sub-paths becomes very minimal. As a result, the envelope of the CIR exhibits a Rayleigh distribution. Considering the locations of the moving vehicle $R_{x}$ and static BS $T_{x}$, the derived CIR of the LOS link can be taken as a deterministic process. If the variables that change with time are introduced into the reference model, the suggested 3D model is capable of describing the non-stationary properties of the HST propagation channels.

### 2.1. Enhanced Gauss–Markov Mobility Model

In the case of the enhanced Gauss–Markov mobility model from [26],$v_{R}\left(t\right)$ represents the velocity of the HST at any time *t*. Therefore, it is possible to represent the non-stationary velocity of the HST at time instant *t* as

(5) $$v_{R}\left(t\right)=Y\left(v_{R}\left(t-\Delta_{t}\right)+\alpha_{R}\Delta_{t}\right)+\left(1-Y\right)\left(\alpha_{R}t+v_{R}\left(t_{0}\right)\right)+\sqrt{1-Y_{\chi_{t-\Delta_{t}}}},$$

In this case, $v_{R}\left(t_{0}\right)$ represents the initial velocity of the HST at $t_{0}$. The time separation and the acceleration of the HST are represented as $\Delta_{t}$ and $\alpha_{T}$, respectively. At any time instant t−Δt, we shall have $v_{R}\left(t-\Delta_{t}\right)$. The zero-means Gaussian distribution will represent the variable $\chi_{t-\Delta_{t}}$, which is also random. This will have a variance $\sigma_{v}$. The range [0,1] of Y determines the timeline of the movement of the HST. This movement presents the changing time instant *t* and the preceding time, especially whenever Y is zero.

### 2.2. Cluster Process Evolution

In a time-variant (i.e., non-stationary) environment, clusters can only be present for a short while. With time, new taps constantly appear, survive for a while, or “survive”, and then finally disappear, or “die”. Discrete Markov systems provide a reasonable description for this generation-recombination pattern. The mobility of the MRS in a network design supported by relays is the leading cause of the time variance of the HST communication system. When modeling the clusters of the MPCs, a genetic appearance (birth) and disappearance (death) mechanism has been popularly used to describe changing clusters. A Markov process in [28] was used for representing the time-varying cluster M(t) formation in the suggested model. The time-varying clusters can be represented as

(6) $$EM_{\left(t\right)}=\frac{\lambda_{G}}{\lambda_{R}}$$

The symbols $\lambda_{G}$ and $\lambda_{R}$ represent the clusters’ rates of birth/generation and death/ recombination. In addition, the fluctuations caused by the movement of the MRS can also be derived as

(7) $$\delta \left(t,\Delta t\right)=\int_{t}^{\left(t+\Delta t\right)}v_{R}\;dt$$

Given that the factor $\delta_{t,\Delta t}$ of the channel fluctuates, the approximation can be represented as

(8) $$\delta \left(t,\Delta t\right)\approx \frac{v_{R}\left(t\right)+v_{R}\left(t+\Delta t\right)}{2}$$

The two types of cluster states were analyzed. These were analyzed during the process of development as time changed from *t* to $t+\Delta_{t}$, i.e., the remaining clusters at time *t* and the new clusters being formed. The following formula can be used to determine the cluster’s survival likelihood from *t* to t+Δt.

(9) $$P_{remain}\left(t,\Delta t\right)=e^{-\lambda_{R}\frac{\delta (t,\Delta t)}{D_{c}}}$$

where the type of situation factor Dc quantifies the correlation coefficient of the scenario movement for the evolving clusters, which can also be called the clustered birth–death process correlation gap. The survival probability is shown to be dependent on the channel fluctuation factor δ(t,Δt). The average number of emerging new clusters throughout the same evolution process:

(10) $$E\left(N_{new},\left(t,\Delta t\right)\right)=\frac{\lambda_{G}}{\lambda_{R}}\left(1-e^{-\lambda_{R}\frac{\delta (t,\Delta t)}{D_{c}}}\right)$$

where E(.) denotes the expectation.

Consider a scenario where the cluster birth–death process is significantly impacted by the movements of HSTs, and Equations (8) and (9) are functions for the MRS velocity $v_{R}$. In general, the survival probability of the cluster increases as $v_{R}\left(t\right)$ decreases, and vice versa. Within the Δt period, λG and λR determine the mean maximum numbers of newly developing clusters.

### 2.3. The HST Time-Varying Distances

The minimum distance between the base station and the truck is denoted by $D_{min}$, and the $D_{proj}$ is the projection distance of $D_{s}\left(t\right)$ on the railway truck, which can be derived as in Figure 3 of [9]. By using trigonometry, $D_{s}\left(t\right)=D_{proj}{\left(t\right)}^{2}+h^{2}$, where $h=H_{BS}-H$ and $D_{proj}\left(t\right)=D_{min}^{2}\left(t\right)+D_{verticle}\left(t\right)$. The vertical distance between the base of the base station and the projection of the mobile relay station at time *t* is denoted by $D_{verticle}\left(t\right)$. The antenna is placed on the rooftop of the train.

### 2.4. Method of Equal Volume and the Proposed Sum of the Sinusoidal Simulation Model

The AoAs and AoDs can be integrally computed to determine the channel’s statistical properties, and the analytical model assuming infinite rays in each scatterer is utilized. Unfortunately, the design of a channel simulator restricts the realization of integral computing. Therefore, the SoS simulation model is constructed by substituting an integral calculation with a summing calculation to make the channel model more applicable. The sum of sinusoidal simulation (SoS) model from [29] introduces discrete AoAs and AoDs, since the variables of integration in the integral computation are AoAs and AoDs, and the remaining parameters are the same as those in the analytical model. The von Mises distribution has been proposed to generate the AoAs and AoDs. This method’s fundamental concept is to use the inverse function of integration to create a set of $\left\{\alpha_{i},\beta_{i}\right\}$ that can satisfy the condition

(11) $$\int_{-\pi }^{\alpha_{i}}\int_{-\pi }^{\beta_{}i}f\left(\alpha ,\beta \right)d\alpha d\beta =\frac{1-1/4}{N_{i}}$$

This method’s comprehensive description is presented in [30]. Based on the MMEV and the suggested model, the corresponding channel characteristics of the SoS simulation model can be produced.

However, to formulate the deterministic simulation model, a fixed number of inter-cluster subpaths with zero phase is assumed. By using the method described of equal areas (MMEV) in [30], the discrete set of $\alpha_{n,m}^{R},\beta_{n,m}^{R},R_{n,m}$ is developed based on these distributions in (11). These new scatterers are consequently dispersed all around the MRS. Then, the other variables, particularly AAoDs and EAoDs, can be determined following the geometric relationship described above. The variables $\alpha_{n,m}^{T}$ and $\beta_{n,m}^{T}$ can be deduced, as we can derive the following relationship between the AoDs and AoAs of the SB rays:

(12) $$\alpha_{n,m}^{T}\approx \frac{R_{n,m}}{D}\sin\alpha_{n,m}^{R}$$

(13) $$\alpha_{n,m}^{T}\approx \arccos\left(\frac{D+R_{n,m}\cos\beta_{n,m}^{R}\cos\alpha_{n,m}^{R}}{\xi_{n,m}^{T}}\right)$$

We continue to derive $\alpha_{n,m}^{T}$ and $\beta_{n,m}^{T}$ from (11) and (12) to understand the development of AAoDs and EAoDs. The basic characteristics of HST channels, such as the time-variant speed, AoA and AoD, power delay profile (PDP), and delay variables, are realized through this development process.

### 2.5. Cluster Delay Update

Once the departure and arrival angles MPC have been defined at a specific moment, The propagation distance for each propagation path from the $T_{x}$ to the $R_{x}$ can be determined. The LOS path’s propagation delay is determined using the following equation:

(14) $$\tau_{Los}\left(t\right)=\frac{\varepsilon_{pq}\left(t\right)}{c}$$

In this case, *c* denotes light speed. In addition, given the $m^{th}$ sub path for $n^{th}$ cluster, the propagation delay is given by

(15) $$\tau_{n,m}\left(t\right)=\varepsilon_{p,S_{nm}}\left(t\right)+\varepsilon_{S_{nm},q}\left(t\right))/c$$

The sub-paths of the propagation delay in (14) become generic for both emerging clusters and survival clusters. The cluster center has to be estimated first to determine a cluster’s delay. The power means, which is denoted by the *K* framework, is commonly employed to evaluate the disparities between MPCs, as in [31]. For this model, the difference between AAoAs of intra-cluster and sub-paths traveling through the center is considered to be not greater than π. The total multipath component distance along a specific sub-path for i∈M(t) with other intra-cluster sub-paths *i* is based on an approximation

(16) $$\rho_{i}=\sum_{i=i}^{M(t)}\sqrt{{\left(\tau_{n,j}-\tau_{n,i}\right)}^{2}\left|+\right|g_{n,j}^{R}-g_{n,i}^{R}{\left|\right|}^{2}}$$

where the multipath component distance angular terms $g_{n,j}^{R}$ and $g_{n,i}^{R}$ are given by $g_{n,j}^{R}=\left[\cos\beta_{n,i}^{R}\cos\alpha_{n,j}^{R},\cos\beta_{n,j}^{R}\sin\alpha_{n,j}^{R},\sin\beta_{n,j}^{R}\right]$

By minimizing (15), the multipath component distances between every sub-path and the rest of the subpaths are determined from minimization. The time of arrival delay of the subpath $\tau_{n,j}$ from the $n^{th}$ cluster with a delay of $\tau_{n}$. The cluster’s power generation is dependent on the WINNER II channel model [18]. The normalized cluster delays indicated by the symbol $\tau_{n}$ are arranged in increasing order.

(17) $$\tau_{n}^{\prime }=sort\left(\tau_{n}-min\left(\tau_{n}\right)\right)$$

### 2.6. Cluster Power Update

Cluster powers are computed with the assumption of a power delay profile with a single slope exponential. The parameters provided by the initial IMT-A channel model can be utilized to compute the randomly mean power for the $n^{th}$ cluster, whereas at time $t_{0}$, the power delay is denoted as $P_{n}\left(t_{0}\right)$, as in [32]. Therefore, using the time-varying delays determined earlier, the random average powers for the $n^{th}$ cluster at time *t* may be represented as The exponential distribution is used for the cluster power, and the normal distribution is for the shadowing per cluster.

(18) $$P_{n}^{\prime }\left(t\right)=e^{\left(-\tau_{n}^{\prime }\frac{r_{\tau }-1}{r_{\tau }\sigma_{\tau }}\right).10^{-\frac{Z_{n}}{10}}}$$

where $\sigma_{\tau }$ denotes delay spread, $Z_{n}$ is the per cluster shadowing, and $r_{\tau }$ is the delay distribution proportionality factor. The sum of the cluster is normalized to one, and thus, the individual cluster powers have to be normalized using

(19) $$P_{n}\left(t\right)=\frac{P_{n}^{\prime }\left(t\right)}{\sum_{n=1}^{N(t)}P_{n}^{\prime }\left(t\right)}$$

The cluster power is modeled in the death–birth process, as in [27], to have a linear increase or decrease towards a threshold value. This is to prevent interruptions during the process. When the cluster power is averaged, the sub-path power is also assumed to be identical. Therefore, the sum of all subpaths is one due to normalization.

## 3. The Statistical Properties of the Channel Models

### 3.1. Local Space-Time Correlation Function

The normalized local complex space-time correlation function (ST-CF) of the suggested model is described as

(20) $$\rho h_{pq,\prime p\prime q}\left(\delta_{T},\delta_{R},t,\Delta t\right)=\frac{E[h_{pq}^{*}\left(t\right)h_{\hat{p}\prime q}\left(t+\Delta t\right)]}{\sqrt{E[|h_{pq}^{*}{\left(t\right)|}^{2}]E[|h_{\prime p\prime q}\left(t+\Delta t){|}^{2}\right]}}$$

In this case, E. represents the expectation operator, and the complex conjugate operator is denoted by ${\left(.\right)}^{*}$. The superposition of the NLOS component and LOS component is used to determine the ST-CF as follows:

(21) $$\rho h_{pq,\prime p\hat{q}}\left(\delta_{T},\delta_{R},t,\Delta t\right)=\rho^{LoS}h_{pq,\prime p\prime q}\left(\delta_{T},\delta_{R},t,\Delta t\right)+\rho^{NLoS}h_{pq,\prime p\prime q}\left(\delta_{T},\delta_{R},t,\Delta t\right)$$

By applying the corresponding distribution,

(22a) $$\rho^{LoS}h_{pq,\prime p\hat{q}}\left(\delta_{T},\delta_{R},t,\Delta t\right)=\frac{E[h_{pq}^{*LoS}\left(t\right)h_{\prime p\prime q}^{LoS}\left(t+\Delta t\right)]}{\sqrt{E[|h_{pq}^{*}{\left(t\right)|}^{2}]E[|h_{\prime p\prime q}\left(t+\Delta t\right){|}^{2}]}}$$

(22b) $$=\sqrt{\frac{K(t)}{K(t)+1}}e^{-j2\pi (\varepsilon_{p,q}\left(t\right)-\varepsilon_{p,q}\left(t+\Delta t\right))/\lambda }e^{j2\pi f_{D}^{Los}\left(t+\Delta t\right)t}\times e^{j2\pi f_{D}^{Los}\left(t+\Delta t\right)\Delta t}\times e^{j2\pi f(\tau_{Los}\left(t\right)-\tau_{Los}\left(t+\Delta t\right))}$$

(23a) $$\rho^{NLoS}h_{pq,\prime p\prime tq}\left(\delta_{T},\delta_{R},t,\Delta t\right)=\frac{E[h_{pq}^{*NLoS}\left(t\right)h_{\prime p\prime q}^{LoS}\left(t+\Delta t\right)]}{\sqrt{E[|h_{pq}^{*}{\left(t\right)|}^{2}]E[|h_{\prime p\prime q}\left(t+\Delta t\right){|}^{2}]}}$$

(23b) $$=\sqrt{\frac{1}{\left(K(t)+1\right)}}\sum_{n=1}^{N(t)}P^{NLos}\lim_{M(t)\to \infty }\frac{1}{M(t)}\sum_{m}^{M(t)}\times e^{j2\pi A^{NLos}}/\lambda \times e^{j2\pi B^{NLos}}\times e^{j2\pi f_{nm,D(t+\Delta t)\Delta t}}\times e^{j2\pi f(\tau_{n,m}\left(t\right)-\tau_{n,m}\left(t+\Delta t\right))}$$

where $P^{NLos}=\sqrt{P_{n}\left(t\right)P_{n}\left(t+\Delta t\right)}$, $A^{NLos}=\varepsilon_{p,S_{nm}}\left(t\right)+\varepsilon_{S_{nm},q}\left(t\right)-\varepsilon_{\prime p,S_{nm}}\left(t+\Delta t\right)-\varepsilon_{S_{nm},q}\left(t+\Delta t\right),B^{NLos}=\left(f_{nm,D(t+\Delta t)}-f_{nm,D(t)}\right)$.

### 3.2. Channel Time-Variant Transfer Function

The Fourier transform of the time-variant CIR is obtained from the time-variant channel transfer function (TCTF) as

(24) $$h_{pq}\left(t,f\right)=h_{pq}^{Los}\left(t,f\right)+h_{pq}^{NLos}\left(t,f\right).$$

where $h_{pq}^{Los}\left(t,f\right)$ and $h_{pq}^{NLos}\left(t,f\right)$ represent the two components i.e., the LOS and NLOS components of the TCTF, defined as

(25a) $$h_{pq}^{Los}\left(t,f\right)=\sqrt{\frac{K(t)}{K(t)+1}}e^{-j2\pi \varepsilon_{p,q}\left(t\right)/\lambda }e^{j2\pi f_{D}^{Los}\left(t\right)t}\times e^{-2j\pi f\tau_{Los}\left(t\right)}$$

(25b) $$h_{pq}^{NLos}\left(t,f\right)=\sqrt{\frac{1}{\left(K(t)+1\right)}}\sum_{n=1}^{N(t)}\lim_{M(t)\to \infty }\sqrt{\frac{P_{n}\left(t\right)}{M(t)}}\times \sum_{m=1}^{M(t)}e^{-j2\pi (\varepsilon_{p,S_{nm}}\left(t\right)+\varepsilon_{S_{nm},q})/\lambda }\times e^{j2\pi f_{mn,D(t)}t}\times e^{j\phi_{n,m}\left(t\right)}\times e^{-2j\pi f\tau_{n,m}\left(t\right)}$$

### 3.3. The RMS Delay Spread

The root-mean-square-delay spread is the measure of wideband delay dispersion which is derived as the square root of the PDP channel in [28]:

(26) $$\sigma_{\tau }=\sqrt{\bar{\tau^{2}}-{\bar{\tau }}^{2}}$$

Whereas the summation of the power-weighted averages of the mean Doppler shifts is derived from the mean Doppler sifts. The time-varying mean Doppler shift at time *t* is denoted by ${\bar{\tau }}^{2}$, and therefore,

(27) $$\bar{\tau }\left(t\right)=\frac{1}{k+1}\left(\sum_{n=1}^{N(t)}\sum_{m=1}^{M(t)}P_{n,m}\left(t\right)f_{nm,D(t)}+Kf_{Los}\left(t\right)\right)$$

For the $m^{th}$ sub-path for $n^{th}$ cluster, the associated power is given by $P_{n,m}\left(t\right)$.

While considering a non-WSS channel, at any time *t*, the mean Doppler shifts $\bar{\tau }\left(t\right)$ are used to derive the time-variant. The root mean square-delay spread denoted by $\sigma_{\tau }\left(t\right)$ is

(28) $$\sigma_{\tau }\left(t\right)=\sqrt{\frac{1}{k+1}}\times \sqrt{\left(\sum_{n=1}^{N(t)}\sum_{m=1}^{M(t)}P_{n,m}\left(t\right)f_{nm,D(t)}+Kf_{Los}\left(t\right)\right)}-{\bar{\tau }}^{2}$$

In the same manner, the azimuth/elevation angular spread of departure and the azimuth/elevation angular spread of arrival can all be calculated with the same procedure.

#### The Stationary Time Interval

To obtain the quasi-stationary interval, in this work the channel is often classified into the WSS and non-WSS, as in [33]. In this paper, a quasi-stationary channel is assumed when the parameter *w*, which is also equal to $\varrho (T_{s})$, is 10%. The mean Doppler shift $\bar{\tau }\left(t\right)$ and RMS-DS $\sigma_{\tau }\left(t\right)$ are both time variants in non-WSS channels. These are assumed to be constant for a WSS channel. The quasi-stationary time interval is used to evaluate the relative error of the RMS-DS across time. The equation shows that the shortest time interval, Ts, is the quasi-stationary time interval, which is described by

(29) $$\varrho \left(T_{s}\right)=\frac{|\sigma_{\tau }\left(t\right)\left(t+T_{s}\right)-\sigma_{\tau }\left(t\right)|}{\sigma_{\tau }\left(t\right)}$$

## 4. Numerical Simulation and Results

The statistical characteristics of the suggested theoretical and simulation models are analyzed and evaluated in this section. Through numerical simulations, we look into how several important model parameters affect the channel characteristics. In this case, some of the numerical values used are in Table 2.

**Figure 2:**
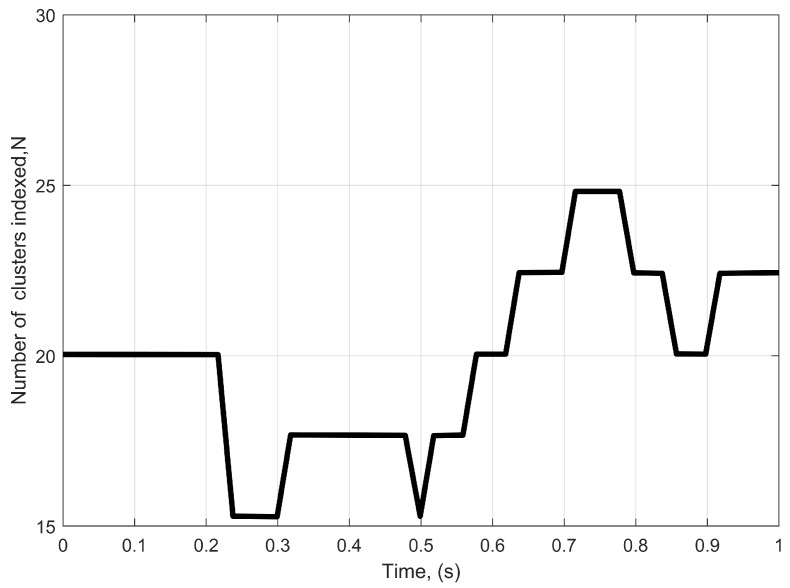
The death–birth process of the total number of clusters versus time.

Figure 2 displays the time-variation of the total number of clusters with the Markov process. In this case, the initial cluster number is set to be $N(t_{0})=20$, which is also used in the IMT-A channel model [34]. From the NLoS UMa environment, the following parameters are considered, where the rate for cluster appearance is $\lambda_{A}$ = 0.8/m and the rate for cluster disappearance is $\lambda_{D}$ = 0.04/m. Then, $P_{s}=0.3,v_{R}$ = 60 m/s.

**Figure 3:**
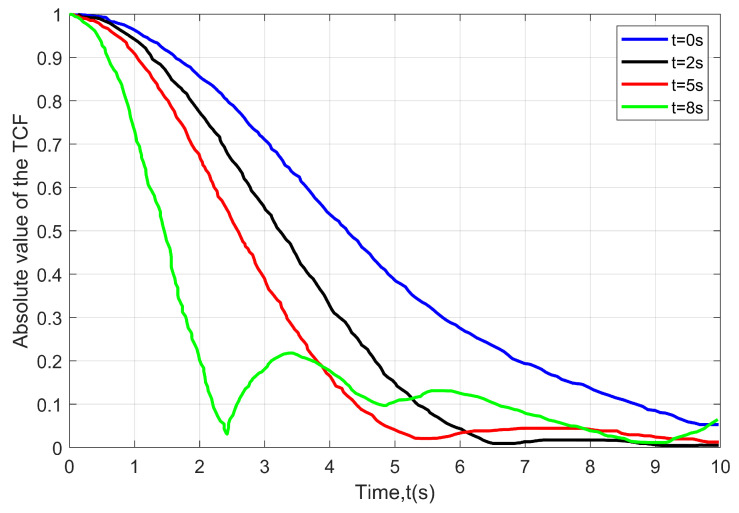
The absolute TCFs at any time instant t(s).

Figure 3 depicts the time-varying absolute value of the TCFs at different instances. It is observed that there is a decrease in time correlation with an increase in time. The suggested model is capable of describing the non-WSS channel, since the time correlation varies for different time instances. Further, another factor that affects the time correlation is changing the angle direction of the MRS and the locations of the scatterers for any given trajectory of the HST. The correlation between different time separations is shown to be time-varying. At time t=0, it has a higher correlation than at other times due to increasing acceleration.

**Figure 4:**
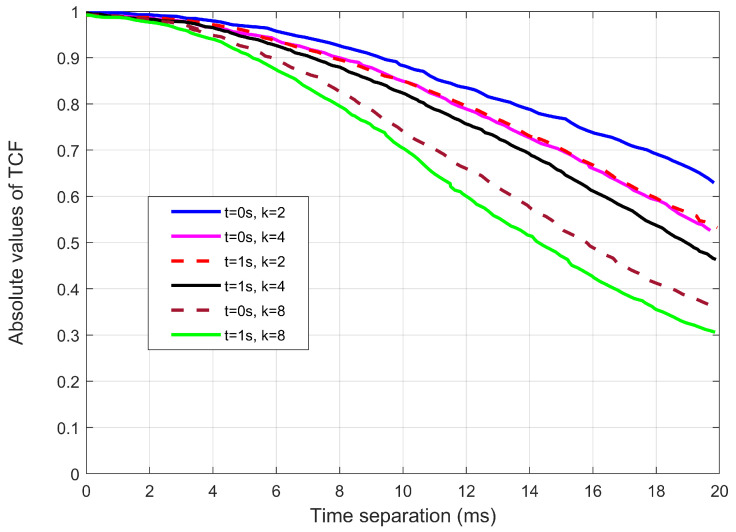
The relationship between the *k* distributions of intra-cluster paths and time correlations for azimuth angles.

Figure 4 shows how the parameter of *k* impacts the AAoA. The absolute value of the TCFs for different *k* is observed. For example, at t=0 s and t=1 s, it is seen that a large *k* causes a higher channel correlation. The explanation is that as *k* increases, the intra-cluster scatterers gradually get more densely concentrated in particular directions.

**Figure 5:**
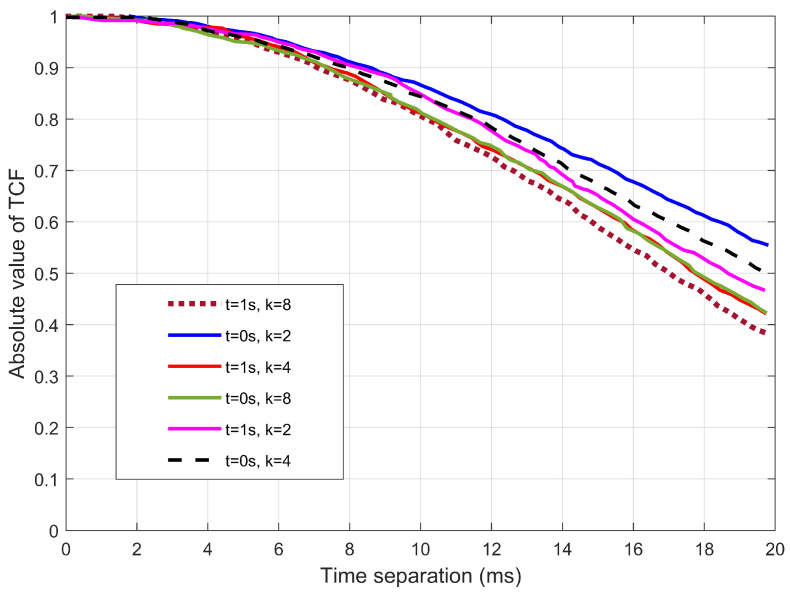
The relationship between the *k* distributions of intra-cluster paths and time correlations for elevation angles.

Figure 5 depicts the absolute value of the TCFs for different angles of elevation. Since the angles were obtained using von misses distribution, and thus the EAoA are dependent on *k*. From Figure 5, there are different time separations required for different parameters of *k*. For example, the figure indicates that for the correlation to drop to 0.5, it requires about 17.5 and 18.5 ms for k=2 and k=8, respectively.

**Figure 6:**
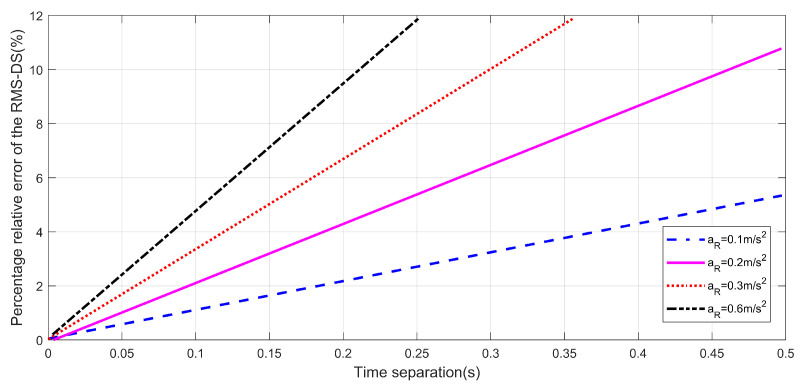
The percentage relative errors of the RMS-DS for various accelerations.

Figure 6 shows the percentage relative error of RMS-DSS for different accelerations $\alpha_{R}$. In addition, the increasing MRS acceleration affects the quasi-stationary time interval by decreasing it. This is a result of the increasing Doppler shift. This indicates that during take-off of the HST, or increasing the acceleration at any point of the trajectory, the channel is more non-stationary. For example, by taking an increase in acceleration of the HST from 0.2 to 0.3 m/s${}^{2}$, it is observed that the quasi-stationary time can decrease by around 0.09s. From Table 3, the quasi-stationary time for varying acceleration is shown. Considering a *w* equal to 10%, the results are shown in Figure 6.

**Figure 7:**
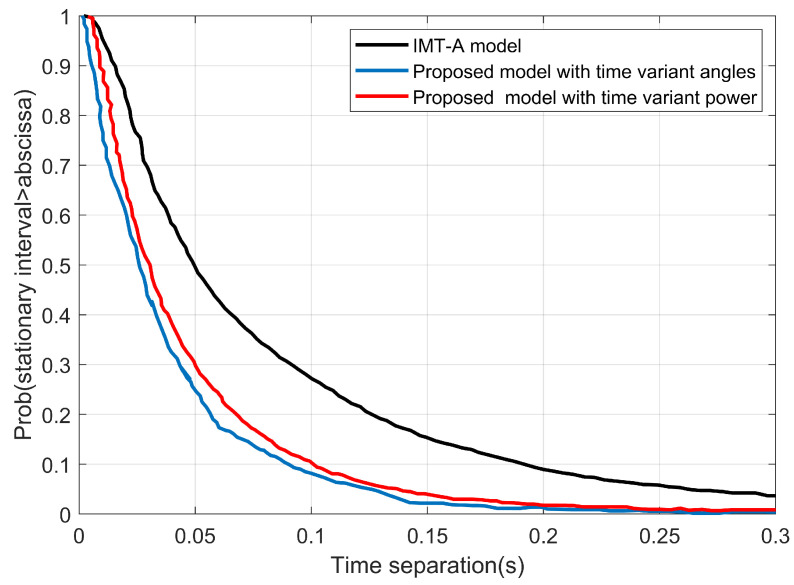
The stationary intervals using the proposed model for time-varying angle, cluster power, and the IMT-A channel.

**Figure 8:**
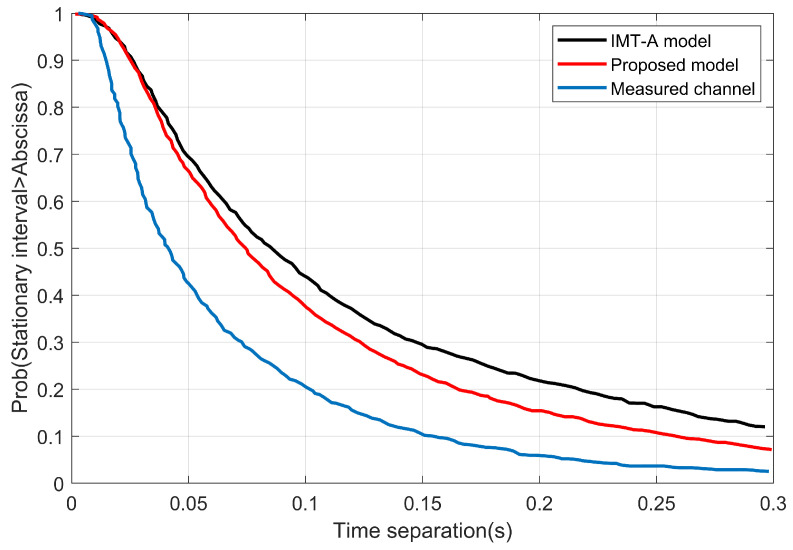
The stationary interval of the proposed model, measured channel, and the IMT-A channel model.

Figure 7 depicts how the angle parameters affect the stationary interval more than the cluster power. Figure 8 shows the stationary interval of the proposed model, which used the simulation parameters that were chosen based on the measurement configuration in [35] and the IMT-A channel model in [34]. As opposed to the measured and the IMT-A channel model, the stationary interval of the proposed model is equal to 14 ms for 80% and 20 ms for 60%. These are significantly shorter compared to the measured data: 9 ms for 80% and 20 ms for 60%. Then, the original IMT-A channel model produced 22.5 ms for 80% and 38.3 ms for 60%.

## 5. Conclusions

A 3D mobility non-stationary cluster-based model has been proposed for the HST channel. The channel statistics, i.e., the space-time correlation function, the root-mean-square Doppler shift, and the quasi-stationary interval were derived from the non-stationary model. The model shows how the quasi-stationary interval decreases with an increasing acceleration of the HST. Compared with the IMT-A advanced channel, the measured data and proposed model’s stationary intervals are significantly shorter. The proposed model has a good agreement with measured results. Furthermore, this interval decreases with increasing acceleration of the HST making it important to incorporate the mobility model in the HST channel. Reason being, the trajectory of the train can be dynamic as it moves along the truck gradient during starting and stopping. For future research, the model can be further extended from MIMO to massive MIMO. This can be considered for higher frequencies in the SAGSIN wireless channel modeling application.

## Figures and Tables

**Table 1:** Definition of parameters.

Parameter	Definition
D	The horizontal distance between the center of the MRS and BS at initial time
$\;R_{\left(n,m\right)}$	radius of the sphere around MRS
$\;f_{s}\left(t\right)$	half spacing between the two foci of the ellipse
$\delta_{T}$,$\delta_{R}$	antenna spacing at the MRS and BS
$\theta_{T}$,$\theta_{R}$	orientation of the MRS and BS antenna array in the x−y plane, respectively
$\varphi^{T}$,$\varphi^{R}$	angles of elevation of the MRS and BS antenna array relative to the x−y plane, respectively
$\;v_{R}$	MRS velocity
$\gamma_{R}$	motion direction of the MRS
$\alpha_{R}^{Los}$, $\beta_{R}^{Los}$	AAoA and EAoA of the Los path, respectively,
$\;S_{\left(n,m\right)}$	The $m^{th}$ scatterer in the $n^{th}$ cluster
$\alpha_{\left(n,m\right)}^{R}$	AAoA of the wave traveling from effective scatterers $\;S_{\left(n,m\right)}$ of the $n^{th}$ cluster
$\alpha_{\left(n,m\right)}^{T}$	AAoD of the wave that impinges from effective scatterers $\;S_{\left(n,m\right)}$ of the $n^{th}$ cluster
$\beta_{\left(n,m\right)}^{R}$	EAoA of the wave traveling from effective scatterers $\;S_{\left(n,m\right)}$ of the $n^{th}$ cluster
$\beta_{\left(n,m\right)}^{T}$	EAoD of the wave that impinges from effective scatterers $\;S_{\left(n,m\right)}$ of the $n^{th}$ cluster
$\gamma_{S},\phi$	Horizontal and elevation moving direction of scatterers, respectively
K	Ricean K factors
$\;V_{S}$	Velocity of moving scatterers

**Table 2:** Numerical values used for the simulation model.

Parameter	Numerical Value
N(t)	20
$\theta_{T}=\theta_{R}$	$45^{\circ }$
$\gamma_{T}=\gamma_{R}$	$0^{\circ }$
$\beta_{T}=\beta_{R}$	$15^{\circ }$
$\alpha_{T}=\alpha_{R}$	$25^{\circ }$
$\alpha_{R}$	4 m/s${}^{2}$
Ψ	0.9
$\sigma_{v}$	0.01
$v_{R}(t_{0})$	250 km/h
$v_{S}$	0.5 m/s
$R_{R}$	50 m
$D_{min}$	50 m
$H_{MRS}$	30 cm
$H_{train}$	3.8 cm
$f_{c}\left(t\right)$	2.6 GHz
$D_{s}$	1000 m
$\delta_{T}$, $\delta_{R}$	λ/2
$\lambda_{A}$	0.8/m
$\lambda_{D}$	0.04/m
$P_{s}$	0.3
*K*	3.8
p=q	2

**Table 3:** Quasi stationary time for varying acceleration.

Scenarios	Acceleration	Quasi Stationary Time
1	0.6 m/s^s^	0.21 s
2	0.3 m/s^s^	0.3 s
3	0.2 m/s^s^	0.451 s

## References

[1] Cheng X., Huang Z., Bai L. (2022). Channel Nonstationarity and Consistency for Beyond 5G and 6G: A Survey. IEEE Commun. Surv. Tutorials.

[2] Cheng N., He J., Yin Z., Zhou C., Wu H., Lyu F., Zhou H., Shen X. (2021). 6G service-oriented space-air-ground integrated network: A survey. Chin. J. Aeronaut..

[3] Liu Y., Wang C.-X., Huang J. (2019). Recent developments and future challenges in channel measurements and models for 5G and beyond high-speed train communication systems. IEEE Commun. Mag..

[4] Ghazal A., Yuan Y., Wang C.-X., Zhang Y., Yao Q., Zhou H., Duan W. (2016). A non-stationary IMT-advanced MIMO channel model for high-mobility wireless communication systems. IEEE Trans. Wirel. Commun..

[5] Zhou L., Luan F., Zhou S., Molisch A.F., Tufvesson F. (2019). Geometry-based stochastic channel model for high-speed railway communications. IEEE Trans. Veh. Technol..

[6] Ghazal A., Wang C.-X., Haas H., Beach M., Mesleh R., Yuan D., Ge X., Chahine M.K. (2012). A non-stationary geometry-based stochastic model for MIMO high-speed train channels. Proceedings of the 2012 12th International Conference on ITS Telecommunications.

[7] Ghazal A., Wang C.X., Ai B., Yuan D., Haas H. (2014). A nonstationary wideband MIMO channel model for high-mobility intelligent transportation systems. IEEE Trans. Intell. Transp. Syst..

[8] Liu Y., Wang C.-X., Lopez C., Ge X. (2017). 3D non-stationary wideband circular tunnel channel models for high-speed train wireless communication systems. Sci. China Inf. Sci..

[9] Assiimwe E., Marye Y.W. (2022). A Stochastic Confocal Elliptic-Cylinder Channel Model for 3D MIMO in Millimeter-Wave High-Speed Train Communication System. Electronics.

[10] Liao C., Xu K., Xie W., Xia X. (2020). 3-D massive MIMO channel model for high-speed railway wireless communication. Radio Sci..

[11] Assiimwe E., Marye Y.W. (2022). A 3D MIMO Channel Model for a High-Speed Train Millimeter Wave Communication System under Cutting and Viaduct Environments. Electronics.

[12] Bian J., Wang C.X., Gao X., You X., Zhang M. (2021). A general 3D non-stationary wireless channel model for 5G and beyond. IEEE Trans. Wirel. Commun..

[13] Imoize A.L., Ibhaze A.E., Atayero A.A., Kavitha K.V.N. (2021). Standard propagation channel models for MIMO communication systems. Wirel. Commun. Mob. Comput..

[14] Liu Y., Wang C.X., Huang J., Sun J., Zhang W. (2018). Novel 3-D nonstationary mmWave massive MIMO channel models for 5G high-speed train wireless communications. IEEE Trans. Veh. Technol..

[15] Lai F., Wang C.X., Huang J., Gao X., Zheng F.C. (2022). A Novel Beam Domain Channel Model for B5G Massive MIMO Wireless Communication Systems. IEEE Trans. Veh. Technol..

[16] Huang Z., Cheng X., Yin X. (2022). A General 3D Non-Stationary 6G Channel Model With Time-Space Consistency. IEEE Trans. Commun..

[17] Wu S., Wang C.-X., Aggoune E.-H.M., Alwakeel M.M., You X.-H. (2017). A general 3-D non-stationary 5G wireless channel model. IEEE Trans. Commun..

[18] Kyösti P., Meinilä J., Hentilä L., Zhao X., Jämsä T., Schneider C., Narandzić M., Milojević M., Hong A., Ylitalo J. WINNER II Channel Models; IST-4-027756; WINNER II D1.1.2, WINNER II Channel Models. D1. 1.2 V1. 2. IST-4-027756 WINNER II. v1.2. http://www.ist-winner.org.

[19] (2009). R M.2135-1.

[20] Trindade S., da Fonseca N.L.S. (2021). Channel Modeling and Characteristics for 6G Wireless Communications. IEEE Netw..

[21] Li J., Niu Y., Wu H., Ai B., Chen S., Feng Z., Zhong Z., Wang N. (2022). Mobility Support for Millimeter Wave Communications: Opportunities and Challenges. IEEE Commun. Surv. Tutor..

[22] Feng L. (2022). A stochastic confocal ellipsoid channel model for high speed railway MIMO communication systems. Phys. Commun..

[23] Zhang Y., Liu Y., Sun J., Wang C.X., Ge X. (2017). Impact of different parameters on channel characteristics in a high-speed train ray tracing tunnel channel model. Proceedings of the 2017 IEEE 85th Vehicular Technology Conference (VTC Spring).

[24] Rasheed I., Hu F., Hong Y.K., Balasubramanian B. (2020). Intelligent vehicle network routing with adaptive 3D beam alignment for mmWave 5G-based V2X communications. IEEE Trans. Intell. Transp. Syst..

[25] Li Y., Wang W., Gao H., Wu Y., Su M., Wang J., Liu Y. (2020). Air-to-ground 3D channel modeling for UAV based on gauss-markov mobile model. AEU-Int. J. Electron. Commun..

[26] Biomo J.-D.M.M., Kunz T., St-Hilaire M. An enhanced gauss-Markov mobility model for simulations of unmanned aerial ad hoc networks. Proceedings of the 2014 7th IFIP Wireless and Mobile Networking Conference (WMNC).

[27] Gutierrez-Diaz-de-Leon C.A., Patzold M. Sum-of-sinusoids-based simulation of flat-fading wireless propagation channels under non-isotropic scattering conditions. Proceedings of the IEEE GLOBECOM 2007—IEEE Global Telecommunications Conference.

[28] Gutierrez R.M., Yu H., Rong Y., Bliss D.W. Time and frequency dispersion characteristics of the UAS wireless channel in residential and mountainous desert terrains. Proceedings of the 2017 14th IEEE Annual Consumer Communications & Networking Conference (CCNC).

[29] Yuan Y., Wang C.X., Cheng X., Ai B., Laurenson D.I. (2014). Novel 3D geometry-based stochastic models for non-isotropic MIMO vehicleto-vehicle channels. IEEE Trans. Wireless Commun..

[30] Zwick T., Fischer C., Didascalou D., Wiesbeck W. (2000). A stochastic spatial channel model based on wave-propagation modeling. IEEE J. Sel. Areas Commun..

[31] Bian J., Sun J., Wang C.X., Feng R., Huang J., Yang Y., Zhang M. (2018). A WINNER+ based 3-D non-stationary wideband MIMO channel model. IEEE Trans. Wireless Commun..

[32] Chang H., Bian J., Wang C.X., Bai Z., Zhou W. (2019). A 3D non-stationary wideband GBSM for low-altitude UAV-to-ground V2V MIMO channels. IEEE Access.

[33] Paetzold M., Gutierrez C.A. Definition and analysis of quasistationary intervals of mobile radio channels-invited paper. Proceedings of the 2018 IEEE 87th Vehicular Technology Conference (VTC Spring).

[34] Baum D.S., Kyösti P., Meinilä J., Jämsä T. Final report on link level and system level channel models. Proceedings of the Wirless World Research Forum Meeting 15.

[35] Zhang J. (2019). IMT-2020 Channel Model. Wiley 5G Ref: The Essential 5G Reference Online.

